# A multi-locus genome-wide association study reveals the genetics underlying muscadine antioxidant in berry skin

**DOI:** 10.3389/fpls.2022.969301

**Published:** 2022-08-04

**Authors:** Minkyu Park, Ahmed G. Darwish, Rashid I. Elhag, Violeta Tsolova, Karam F. A. Soliman, Islam El-Sharkawy

**Affiliations:** ^1^Center for Viticulture and Small Fruit Research, College of Agriculture and Food Sciences, Florida A&M University, Tallahassee, FL, United States; ^2^Department of Biochemistry, Faculty of Agriculture, Minia University, Minia, Egypt; ^3^College of Science and Technology, Florida A&M University, Tallahassee, FL, United States; ^4^College of Pharmacy and Pharmaceutical Sciences, Florida A&M University, Tallahassee, FL, United States

**Keywords:** antioxidant, GWAS, muscadine, total flavonoid content, total phenolic content

## Abstract

Muscadine berries display enhanced nutraceutical value due to the accumulation of distinctive phytochemical constituents with great potential antioxidant activity. Such nutritional and health merits are not only restricted to muscadine, but muscadine berries accumulate higher amounts of bioactive polyphenolics compared with other grape species. For the genetic study of the antioxidant trait in muscadine, a multi-locus genome-wide association study (GWAS) with 350 muscadine genotypes and 1,283 RNase H2 enzyme-dependent amplicon sequencing (rhAmpSeq) markers was performed. Phenotyping was conducted with several antioxidant-related traits, including total phenolic content (TPC), total flavonoid content (TFC), 1,1-diphenyl-2-picrylhydrazyl (DPPH) free radical scavenging activity, and FRAP antioxidant assay in muscadine berry skin. The correlation coefficient analysis revealed that the TPC, and DPPH/FRAP activities were significantly correlated. Through the GWAS analysis, 12 QTNs were identified from the four traits, of which six were pleiotropic QTNs. Two pleiotropic QTNs, chr2_14464718 and chr4_16491374, were commonly identified from the TPC and DPPH/FRAP activities. Co-located genes with the two pleiotropic QTNs were isolated, and two candidate genes were identified with transcriptome analysis. UDP-glycosyltransferase and 4-hydroxy-4-methyl-2-oxoglutarate aldolase were the candidate genes that are positively and negatively correlated to the quantitative property of traits, respectively. These results are the first genetic evidence of the quantitative property of antioxidants in muscadine and provide genetic resources for breeding antioxidant-rich cultivars for both *Muscadinia* and *Euvitis* species.

## Introduction

Muscadine grape (*Muscadinia rotundifolia* Michx.) is a native grape of the southeastern United States with great marketing opportunities for juice, wine, and fresh fruit (Olien and Hegwood, 1990; Hoffmann, 2020). *Muscadinia* belongs to the genus *Vitis*, composed of two subgenera, *Euvitis* (bunch grapes) and *Muscadinia* (muscadine grapes). While *Euvitis* has approximately 70 species, only three species, *M. rotundifolia*, *M. munsoniana*, and *M. popenoei* are known in the *Muscadinia* genus (Brizicky, 1965; Zecca et al., 2020). Among the three *Muscadinia* species, only *M. rotundifolia* is commercially cultivated.

Muscadine grapes are gained more popularity recently with their distinctive flavor and aroma properties and are an important source of essential oils, vitamins, minerals, fiber, nutraceutical compounds, and antioxidants (Olien, 1990; Ector et al., 1996; Pastrana-Bonilla et al., 2003; Yilmaz and Toledo, 2004; Stringer et al., 2009; Alkan et al., 2021; Kupe et al., 2021; Taskesenlioglu et al., 2022). In particular, the health benefits of muscadine grapes due to phenolic compounds were reported for anticancer (Hudson et al., 2007), anti-cardiovascular diseases (Mellen et al., 2010), and anti-microbial (Xu et al., 2014) effects. The muscadine berries are mainly consumed with fresh fruit, juice, jam, and wine. In these products, the skin and pulp are mainly consumed. Therefore, the phenolic content in the skin and pulp are important to increase the marketability of muscadine grapes.

The accumulation of total phenolic compounds in muscadine berries is highest in seeds, followed by skin and pulp (Sandhu and Gu, 2010). It is reported that muscadine berry skin contains about seven times higher phenolic content than pulp (Darwish et al., 2021). Therefore, increasing the phenolic content in the skin is an important breeding target of the muscadine grape. Many reports have been on muscadine grapes related to the profiling of phenolic compounds and antioxidant capacities (Pastrana-Bonilla et al., 2003; Sandhu and Gu, 2010). However, no genetic study related to those traits has been reported. Considering the importance of the trait in muscadine grapes, the genetic study of this trait is highly required to develop molecular markers for breeding and selection.

Breeding perennial crops like muscadine grape are expensive and time-consuming due to the large plant size and long juvenile phase (Migicovsky and Myles, 2017). One efficient approach to reducing the cost and time is developing marker-assisted selection (MAS). The availability of the genome sequence of the muscadine grape provides efficient resources to develop molecular markers for muscadine breeding. The chromosome-level whole-genome reference sequences have recently been released in two muscadine cultivars, “Noble” and “Trayshed” (Cochetel et al., 2021; Park et al., 2022). Based on these genome sequences, a genome-wide association study (GWAS) of muscadine was conducted in our previous study, and 12 berry-related traits were identified (Park et al., 2022). To use the MAS in muscadine breeding, further efforts are needed to develop the agronomic trait-associated markers. In this study, we performed a multi-locus GWAS analysis to identify the loci associated with the quantitative property of total phenolic and flavonoid content and their antioxidant capacities.

## Materials and methods

### Plant materials

A total of 350 muscadine genotypes were used for phenotyping and genotyping to conduct the GWAS analysis. All the muscadine individuals were grown at the experimental vineyard of the Florida A&M University (Tallahassee, FL, United States). The DNA for genotyping was extracted from the young leaves of each individual using Qiagen DNeasy Plant Mini Kit (Qiagen, Valencia, CA, United States).

### Phenotyping

Phenotyping of the antioxidant-related traits was evaluated in muscadine berry skin at the maturation stage. The maturation level of berries was determined *via* measuring berry firmness, total soluble sugar (TSS), acidity (TA), and TSS/acid ratio as described previously (Campbell et al., 2021). Five clusters/replicate and three biological replicates/genotype were randomly collected for each individual. The berry skin was carefully separated, immediately frozen in liquid nitrogen, and stored at –80°C for further analysis. All samples were lyophilized, finely ground, and ∼12 g of powder tissues were homogenized in 100 ml of methanol supplemented with 1% HCl. All extractions were performed by shaking (150 rpm) for 24 h/20°C in the dark. All extracts were filtered, supernatants were dehydrated, and dry extracts were stored at 4°C in the dark. The stock solution of skin extracts was prepared at 10 mg/ml in DMSO to determine the total metabolite content and antioxidant activities. The assays of total phenolic content (TPC), total flavonoid content (TFC), 1,1-diphenyl-2-picrylhydrazyl (DPPH) radical-scavenging activity, and ferric reducing antioxidant potential (FRAP) were performed as described previously (Darwish et al., 2021). TPC was expressed as milligram gallic acid equivalents per gram of sample dry weight (mg GAE/g DW), TFC as milligram quercetin equivalents per gram of sample dry weight (mg QE/g DW), DPPH as the percentage scavenging of DPPH radicals (%), and FRAP as micro-molar Trolox equivalents per gram of sample dry weight (μM TE/g DW). The Pearson model was used to calculate the correlation coefficiency among the four phenotypes with pairwise comparison. The result was plotted with “corrplot” package of R software (v4.1.0).

### Genotyping and genome-wide association study analysis

The genotyping of the 356 individuals was performed with a total of 2,000 RNase H2 enzyme-dependent amplicon sequencing (rhAmpSeq) markers (Zou et al., 2020). Filtering and imputation of the markers were conducted as described previously (Park et al., 2022), leaving 1,283 markers that were valid for GWAS analysis.

The population structure of the individuals was analyzed in our previous study, and the number of subpopulations (*k*) was identified as 15 (Park et al., 2022). The Q-matrix for the GWAS was generated with STRUCTURE software using the *k* = 15 option.

We used a multi-locus GWAS method for the sensitive identification of quantitative trait nucleotides (QTNs) (Zhang et al., 2019). A total of six multi-locus GWAS methods, including mrMLM (Wang et al., 2016), ISIS EM-BLASSO (Tamba et al., 2017), pLARmEB (Zhang et al., 2017), FASTmrEMMA (Wen et al., 2018), pKWmEB (Ren et al., 2018), and FASTmrMLM (Tamba and Zhang, 2018) were used to compare the results. One year of the phenotyping data was used in the GWAS analysis. The associations between phenotypes and markers were analyzed with the six methods implemented in the “mrMLM.GUI” package of the R software (v4.1.0) (Zhang et al., 2020).

### Transcriptome analysis

For transcriptome analysis, muscadine berry samples were collected by three replications from three different genotypes, C5-9-2 (“Ison × Fry”), C6-10-1 (“Southland × Fry”), and Late Fry, at three different berry developmental stages, “Fruit-Set,” “Véraison,” and “Ripe.” These genotypes were selected according to their diversity in TPC, TFC, and antioxidant capacities (Darwish et al., 2021; Ismail et al., 2022). The RNA was extracted from the skin as described previously (Ismail et al., 2022). The sequencing library was constructed with NEBNext Ultra II RNA Library Prep Kit for Illumina (New England Biolabs, Ipswich, MA), and sequencing was performed by paired-end 150 bp read in two lanes using NovaSeq 6000 (Illumina, San Diego, CA) at the Novogene Co., Ltd. (Sacramento, CA). The quality of RNA-seq libraries was checked and trimmed with FastQC (v0.11.9). After quality control, the sequences were mapped to the muscadine transcriptome (Park et al., 2022) using the RSEM pipeline (Li and Dewey, 2011). Gene expression levels were calculated and normalized by reads per transcript per kilobase million mapped reads (TPM). The average TPM of the three replicates was used for further analysis.

### Correlation between antioxidant traits and expression of marker-associated genes

The Pearson correlation test was used to calculate the correlation between antioxidant contents and expression of marker-associated genes (*p* ≤ 0.05 and *r* ≥ 0.7 or *r* ≤ –0.7). The TPC, TFC, DPPH activity, and FRAP activity values during berry development were compared to the TPM expression values of the marker-associated genes at the corresponding developmental stages. The genes that significantly correlated to the antioxidant-related traits were identified and used for further analysis.

## Results and discussion

### Phenotyping of antioxidant-related traits in mature muscadine berries

TPC and TFC were measured in 348 individual genotypes, and DPPH and FRAP activities were in 356 (Supplementary Tables 1–4). The average TPC and TFC levels among the population were 52.0 ± 1.0 mg GAE/g DW and 7.8 ± 0.2 mg QE/g DW, respectively (Table 1). Both traits exhibited a wide range among the population, estimated at 151.3 mg GAE/g DW (22.5 ± 1.1 to 173.8 ± 1.5 mg GAE/g DW) and 36.5 mg QE/g DW (3.4 ± 0.1 to 39.9 ± 1.1 mg QE/g DW) for TPC and TFC, respectively. The average DPPH and FRAP activity levels among the population were 12.3% ± 0.7 and 246.6 ± 5.1 μM TE/g DW, respectively. Similarly, the antioxidant activities displayed a wide range among the population, estimated at 83.2% (0.05% ± 0.03 to 83.2% ± 2.2) and 700.8 μM TE/g DW (75.2 ± 1.2 to 776.0 ± 14.2 μM TE/g DW) for DPPH and FRAP, respectively. Based on the average levels of antioxidant-related traits among the population, 39.4 and 35.6% presented high TPC and TFC levels, respectively. At the same time, 38.2 and 41.3% of the population exhibited high DPPH and FRAP antioxidant capacities, respectively. Interestingly, the two genotypes, O15-17-1 and Noble, were categorized among the top genotypes exhibiting the highest TPC levels and antioxidant capacities (Supplementary Tables 1, 3, 4), suggesting the potential involvement of TPC character in coordinating antioxidant capacity traits.

**TABLE 1:** Summary of the antioxidant-related traits.

	Sample no.	Average	Min	Max	*SD* *
TPC (mg GAE/g DW)	350	52.2	22.5	173.8	18.9
TFC (mg QE/g DW)	350	7.8	3.4	39.9	3.7
DPPH (%)	350	12.4	0.1	83.2	13.4
FRAP (μM TE/g DW)	350	246.9	75.2	776	96.7

*SD, Standard Deviation.

### Correlation coefficient analysis among antioxidant-related traits

**FIGURE 1:**
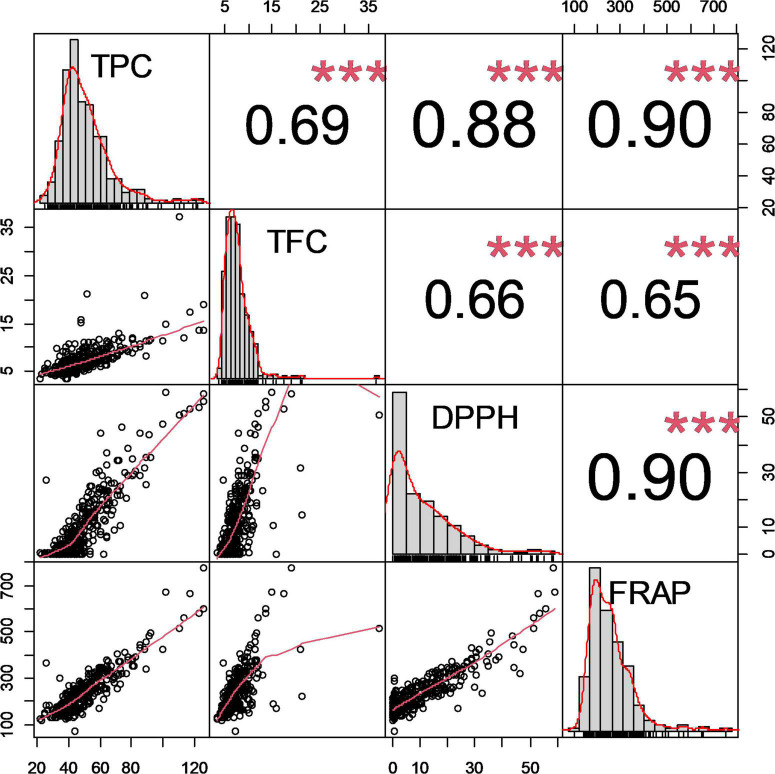
Pairwise correlation coefficient analysis of the antioxidant-related traits. Numbers in the upper diagonal represent the pairwise correlation coefficient value of the antioxidant-related phenotypes. The three stars (***) indicate a significant difference at *p* < 0.001. The graphs on the diagonal represent the distribution histogram of the phenotypes. The graphs in the lower diagonal represent the pairwise distribution of the four antioxidant-related phenotypes.

To investigate the relationship between the four antioxidant-related phenotypes, we conducted correlation coefficient analysis by Pearson correlation test (Figure 1). It is known that TPC and TFC contribute to antioxidant capacities (Silva and Sirasa, 2018). Muscadine TPC showed significant correlations with DPPH (*r* = 0.89; *p* = 1.2 × 10^–118^) and FRAP (*r* = 0.87; *p* = 2.8 × 10^–112^) activities. In contrast, relatively lower, but significant, correlation values of TFC with TPC (*r* = 0.62; *p* = 1.8 × 10^–38^), DPPH (*r* = 0.63; *p* = 9.0 × 10^–40^), and FRAP (*r* = 0.59; *p* = 1.0 × 10^–34^) was observed. It is clear that both TPC and TFC traits contribute to the ultimate antioxidant capacity of muscadine grapes. However, the input of TPC seems to be more significant.

### Genome-wide association study of total phenolic content, total flavonoid content, DPPH, and ferric reducing antioxidant potential

We conducted GWAS analysis with the same muscadine population and genotyping data that were used in our previous study (Park et al., 2022). A total of 350 muscadine genotypes with multiple parent sets were used for genotyping. The genotyping was performed with rhAmpSeq markers, which were developed for marker transferability among *Euvitis* species (Zou et al., 2020). The 2000-rhAmpSeq markers were applied to the muscadine population, obtaining a total of 1,283 markers after filtering.

**FIGURE 2:**
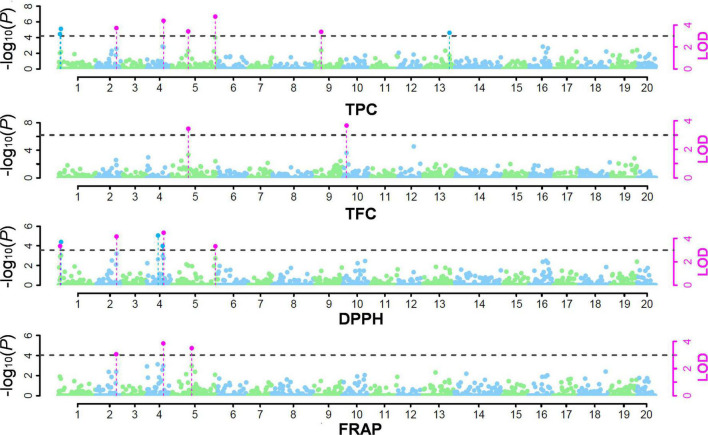
GWAS results for the antioxidant-related traits. The dotted black lines indicate the significance threshold at LOD = 3.0. The significant QTNs detected by multiple and single GWAS methods are presented with pink and blue dots with vertical dotted lines, respectively. For the results by multiple methods, the median value of -log_10_(*P*) from the mrMLM, FASTmrMLM, FASTmrEMMA, and pKWmEB methods was used in plotting.

We used multi-locus GWAS methods because the multi-locus GWAS is known to be more powerful than the single-locus method in detecting QTNs for complex traits (Khan et al., 2019, 2021; Zhang et al., 2019; Muhammad et al., 2021). Through the multi-locus GWAS analysis, a total of 12 QTNs were identified from the four antioxidant-related traits (Figure 2 and Table 2). Three QTNs, chr4_16491374, chr5_24109446, and chr2_14464718, were identified from all six methods (Table 2), suggesting highly reliable QTNs. In contrast, a total of six QTNs were identified by a single method. Among the 12 QTNs, six were pleiotropic QTNs associated with two or more antioxidant-related traits (Table 2, bold names).

**TABLE 2:** List of significant QTNs for the traits identified by the six multi-locus GWAS.

Trait ID	Trait name	QTNs*	Chr.	Position (bp)	QTN effect	LOD score	-log_10_(P)	PVE** (%)	Methods***					
									1	2	3	4	5	6
**1**	TPC	**chr1_547952**	1	431,256	–4.36	3.17	3.87	5.38				•		
		**chr1_1170229**	1	1,001,406	–5.08	3.65	4.38	6.71		•				
		**chr2_14464718**	2	14,729,346	6.24	3.90	4.64	5.27	°	°		°	°	•
		**chr4_16491374**	4	15,299,237	8.62	5.81	6.64	3.09	°	°	°	•	°	°
		**chr5_5884957**	5	4,895,628	9.89	3.70	4.44	17.51	°	°		°	•	°
		**chr5_24109446**	5	19,491,114	7.68	5.69	6.51	2.38	•	°	°	°	°	°
		chr9_2302342	9	2,096,834	5.78	3.63	4.37	8.46		°		•		
		chr13_24703210	13	19,468,259	3.64	3.28	4.00	10.18					•	
**2**	TFC	**chr5_5884957**	5	4,895,628	2.61	3.46	4.18	13.38		•		°		
		chr10_1265993	10	976,118	–2.66	3.84	4.59	3.63		°	°	•		°
**3**	DPPH	**chr1_547952**	1	431,256	–5.15	3.35	4.07	12.37	•	°				°
		**chr1_1170229**	1	1,001,406	–4.05	3.70	4.44	6.86				•		
		**chr2_14464718**	2	14,729,346	6.03	4.50	5.28	8.25	•	°	°	°	°	°
		chr4_9358331	4	7,904,506	–5.37	4.25	5.02	11.70				•		
		chr4_14964966	4	13,429,223	–4.22	3.36	4.08	9.89						•
		**chr4_16491374**	4	15,299,237	–6.71	4.63	5.41	18.92	•	°	°		°	
		**chr5_24109446**	5	19,491,114	4.93	3.50	4.23	1.65	•	°	°		°	°
**4**	FRAP	**chr2_14464718**	2	14,729,346	25.76	3.06	3.76	3.56		•				°
		**chr4_16491374**	4	15,299,237	–35.90	3.86	4.61	12.80		•		°		•
		chr5_7260172	5	6,140,552	–60.25	3.76	4.49	3.64	°	°	•	°		°

*QTN names in bold font are pleiotropic QTNs associated with multiple traits.

**PVE: Phenotypic variation of traits explained by each QTN.

***1: mrMLM; 2: FASTmrMLM; 3: FASTmrEMMA; 4: pLARmEB; 5: pKWmEB; 6: ISIS EM-BLASSO.

•, The most significant method; °, Significant methods.

A total of eight associated QTNs were identified for the TPC trait, of which six among them were pleiotropic QTNs. The TPC and DPPH activity traits shared five of the six pleiotropic QTNs, demonstrating the main contribution of TPC character to the DPPH antioxidant capacity. According to the correlation coefficient analysis, TPC, DPPH activity, and FRAP activity were significant to each other (0.88 ≤ *r* ≤ 0.90). Interestingly, two QTNs, chr2_14464718, and chr4_16491374, were identified as pleiotropic QTNs for these three antioxidant-related traits. In the case of the chr2_14464718 QTN, it was identified with five methods in TPC and six methods in DPPH activity. This indicates that these two QTNs might be highly associated with the antioxidant capacity of muscadine berry skin. For TFC, two associated QTNs were identified, and one of them was a pleiotropic QTN that was also identified in TPC.

### Identification of candidate genes by transcriptome analysis

To identify candidate genes associated with the markers, we investigated the genes between the flanking markers of the target marker from the genome data of *M. rotundifolia* cv. Noble (Park et al., 2022). Because the used muscadine GWAS population was composed of multiple breeding populations with multiple parent sets, the linkage disequilibrium (LD) decay to half of the initial value of the population was observed at 2.3 Mb (Park et al., 2022). Due to the low GWAS resolution, a total of 732 genes were identified with the 12 markers (Supplementary Table 5).

To reduce the number of candidate genes, we performed RNA-seq analyses using three different developmental stages of muscadine berry, Fruit-Set, Véraison, and Ripe. In addition, to use the variation among different genotypes, we also performed the RNA-seq analyses using three different genotypes, C5-9-2, C6-10-1, and Late Fry.

**FIGURE 3:**
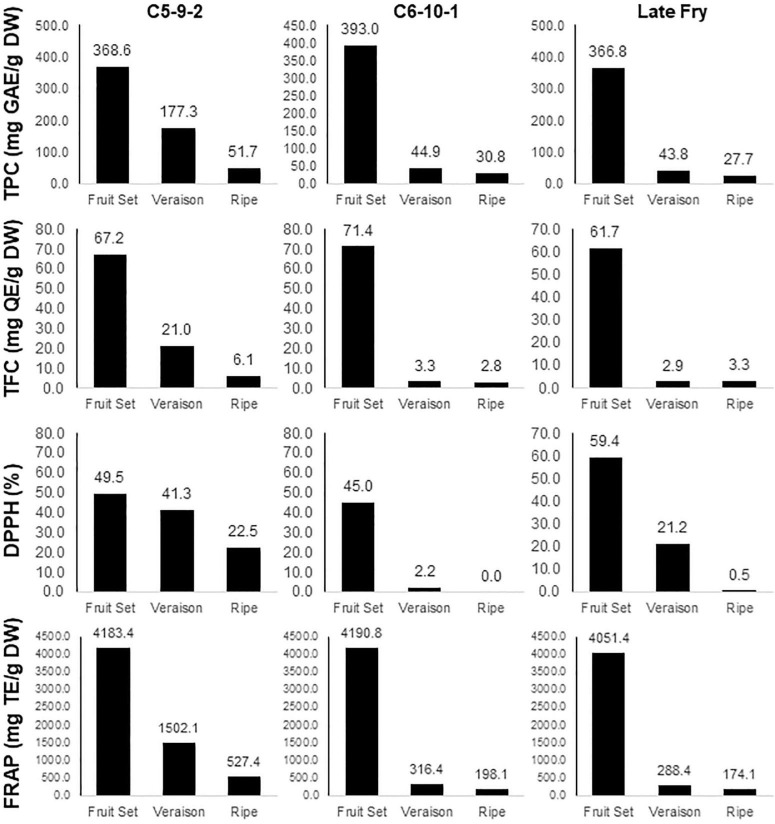
Estimation of TPC, TFC, and DPPH/FRAP activities in the three muscadine genotypes during berry development. The name of the trait and unit of value is presented on the left side of the panel. The name of the genotype is presented in the top.

The phenolic compounds are known not just to accumulate in plant tissues but are subject to rapid turnover and degradation (Barz and Hoesel, 1979). Similarly, our data also showed a rapid decline in the TPC, TFC, and DPPH/FRAP activities along with the progression in berry development (Figure 3). The profile of TPC, TFC, and FRAP traits among developmental stages showed a similar pattern in the three genotypes. While the values in the C5-9-2 genotype were gradually decreased, those in C6-10-1 and Later Fry exhibited a rapid decline of the levels in the Véraison stage and maintained similar values during ripening. In the case of DPPH, contrarily from other traits, the Late Fry showed a gradual reduction in the activity. These results indicate that the quantity of the total phenolic and flavonoid compounds in each developmental stage might be determined by the expression level of the related genes.

### Candidate genes positively correlated to the antioxidant values

**FIGURE 4:**
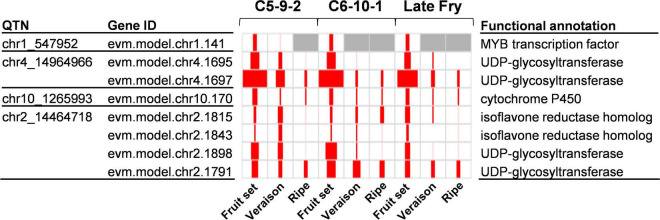
Expression of candidate genes identified by chr2_14464718. The QTN name and corresponding gene ID are listed on the left side of the panel. The functional annotation information is listed on the right side of the panel. In the panel, the size of red squares represents the TPM value of the corresponding gene. The max TPM was 148.8.

To identify the genes that have a significant positive correlation between the expression levels and the values of the four traits, we conducted a Pearson correlation test. The gene expression levels of the 732 candidate genes were compared to the TPC, TFC, and DPPH/FRAP activities from the three genotypes during development. As a result, a total of 145 significantly correlated genes were identified (*r* ≥ 0.7; *p* ≤ 0.05) (Supplementary Table 6). To further reduce the number of candidate genes, we inspected the function of the genes based on the UniProt^1^ and KEGG^2^ database. Accordingly, we could identify eight genes related to the antioxidant activity (Figure 4). Among the eight genes, four encoded UDP-glycosyltransferases, two isoflavone reductase homologs, and the rest were MYB transcription factors and cytochrome P450. Among the candidate genes, UDP-glycosyltransferase was also identified as a strong candidate gene from the association mapping of antioxidants in pearl millet and barley by GWAS (Han et al., 2018; Yadav et al., 2021). This indicates that the UDP-glycosyltransferase of muscadine might also contribute to the quantitative properties of antioxidant capacity. The UDP-glycosyltransferase proteins are known to involve in the synthesis of anthocyanins and flavonoids (Bowles et al., 2005).

UDP-glycosyltransferases were identified by two QTNs, chr4_14964966, and chr2_14464718. In particular, the chr2_14464718 QTN had two copies of UDP-glycosyltransferase. The chr2_14464718 QTN was also co-located with isoflavone reductase homolog genes with an expression pattern significantly correlated with the antioxidant activity (*r* ≥ 0.7; *p* ≤ 0.05). The isoflavone reductase protein is also known to involve the elaboration of flavonoid and isoflavonoid skeletons (Dixon and Steele, 1999). While the chr4_14964966 was identified only by DPPH activity with a single method (ISIS EM-BLASSO), the chr2_14464718 QTN was identified by TPC, DPPH, and FRAP traits, which are significantly correlated. In addition, multiple methods, five in TPC, six in DPPH activity, and two in FRAP activity, identified the chr2_14464718 QTN. Therefore, the chr2_14464718 QTN was the highly probable QTN associated with the antioxidant activity of muscadine berry skins.

Because the UDP-glycosyltransferase and isoflavone reductase homolog genes were identified as multi-copy genes (Supplementary Table 6), we investigated whether there are paralogous genes in the genomic region between the two flanking markers of chr2_14464718. Interestingly, a total of 10 copies of isoflavone reductase homologs and two copies of UDP-glycosyltransferases were identified. There were also 10 MYB transcription factors, but none of them showed a correlation with the antioxidant values. To determine if the copy number of the two candidate genes is related to the quantitative properties of the antioxidant, we investigated the homologous genes in the collinear genomic region of *M. rotundifolia* cv. Trayshed (Cochetel et al., 2021). The Trayshed genome had one UDP-glycosyltransferase and four isoflavone reductase homologs in the collinear region, which were half or less than half of the numbers identified in the Noble cultivar (Table 3). The number of MYB transcription factors in this region was identical between Noble and Trayshed. The Noble cultivar used in this study showed high antioxidant activities in berry skins and ranked as the second in TPC, the sixth in TFC, the third in DPPH activity, and the first in FRAP activity among all the investigated muscadine individuals (Supplementary Tables 1–4). In contrast, the Trayshed is a cultivar that does not bear berries because it produces only male flowers (Massonnet et al., 2020). Therefore, the homologs in the Trayshed cultivar are not functional in berries. The copy number variation of the two candidate genes between the two cultivars might suggest the possible role of the paralogs in the quantitative properties of antioxidants, but further studies are needed to confirm the hypothesis.

**TABLE 3:** Paralogs of UDP-glycosyltransferase and isoflavone reductase homolog co-located with the chr2_14464718 QTN in the Noble genome and their homologs in the collinear region of the Trayshed genome.

	Gene ID	Strand	Start	End	Functional annotation
*M. rotundifolia* cv. Noble	evm.model.chr2.1791	+	13,877,486	13,878,857	**UDP-glycosyltransferase**
	evm.model.chr2.1813	+	14,016,417	14,019,622	Isoflavone reductase homolog
	evm.model.chr2.1814	+	14,026,753	14,029,737	Isoflavone reductase homolog
	evm.model.chr2.1815	+	14,037,454	14,041,047	Isoflavone reductase homolog
	evm.model.chr2.1821	–	14,074,126	14,075,948	Isoflavone reductase homolog
	evm.model.chr2.1822	–	14,078,657	14,080,208	Isoflavone reductase homolog
	evm.model.chr2.1823	–	14,088,843	14,090,433	Isoflavone reductase homolog
	evm.model.chr2.1824	–	14,092,954	14,094,523	Isoflavone reductase homolog
	evm.model.chr2.1826	–	14,103,232	14,105,216	MYB transcription factor
	evm.model.chr2.1831	–	14,115,446	14,115,941	Isoflavone reductase homolog
	evm.model.chr2.1841	–	14,144,693	14,146,300	Isoflavone reductase homolog
	evm.model.chr2.1843	–	14,163,343	14,164,876	Isoflavone reductase homolog
	evm.model.chr2.1844	–	14,168,872	14,172,803	MYB transcription factor
	evm.model.chr2.1865	–	14,283,732	14,284,676	MYB transcription factor
	evm.model.chr2.1877	–	14,349,442	14,350,374	MYB transcription factor
	evm.model.chr2.1879	–	14,376,942	14,377,933	MYB transcription factor
	evm.model.chr2.1881	–	14,381,564	14,382,799	MYB transcription factor
	evm.model.chr2.1884	–	14,396,635	14,397,634	MYB transcription factor
	evm.model.chr2.1898	+	14,474,820	14,480,100	UDP-glycosyltransferase
	evm.model.chr2.1902	–	14,496,908	14,497,892	MYB transcription factor
	evm.model.chr2.1907	–	14,562,145	14,566,273	MYB transcription factor
	evm.model.chr2.1910	–	14,610,052	14,611,030	MYB transcription factor
*M. rotundifolia* cv. Trayshed	VITMroTrayshed_v2.0.hap1.chr02.ver2.0.g024900	–	14,035,820	14,037,812	UDP-glycosyltransferase
	VITMroTrayshed_v2.0.hap1.chr02.ver2.0.g024830	+	13,791,311	13,824,690	Isoflavone reductase homolog
	VITMroTrayshed_v2.0.hap1.chr02.ver2.0.g024820	+	13,776,693	13,780,893	Isoflavone reductase homolog
	VITMroTrayshed_v2.0.hap1.chr02.ver2.0.g024780	+	13,725,066	13,726,672	Isoflavone reductase homolog
	VITMroTrayshed_v2.0.hap1.chr02.ver2.0.g024770	+	13,706,376	13,707,997	Isoflavone reductase homolog
	VITMroTrayshed_v2.0.hap1.chr02.ver2.0.g024760	+	13,698,954	13,702,483	MYB transcription factor
	VITMroTrayshed_v2.0.hap1.chr02.ver2.0.g024730	+	13,577,224	13,577,605	MYB transcription factor
	VITMroTrayshed_v2.0.hap1.chr02.ver2.0.g024710	+	13,531,590	13,533,655	MYB transcription factor
	VITMroTrayshed_v2.0.hap1.chr02.ver2.0.g024700	+	13,449,251	13,470,101	MYB transcription factor
	VITMroTrayshed_v2.0.hap1.chr02.ver2.0.g024680	+	13,420,728	13,422,978	MYB transcription factor
	VITMroTrayshed_v2.0.hap1.chr02.ver2.0.g024640	+	13,377,421	13,378,652	MYB transcription factor
	VITMroTrayshed_v2.0.hap1.chr02.ver2.0.g024590	+	13,322,931	13,330,264	MYB transcription factor
	VITMroTrayshed_v2.0.hap1.chr02.ver2.0.g024580	+	13,299,249	13,300,005	MYB transcription factor
	VITMroTrayshed_v2.0.hap1.chr02.ver2.0.g024570	+	13,294,205	13,295,438	MYB transcription factor
	VITMroTrayshed_v2.0.hap1.chr02.ver2.0.g024560	+	13,239,757	13,240,991	MYB transcription factor

### Candidate genes negatively correlated to the antioxidant values

**FIGURE 5:**
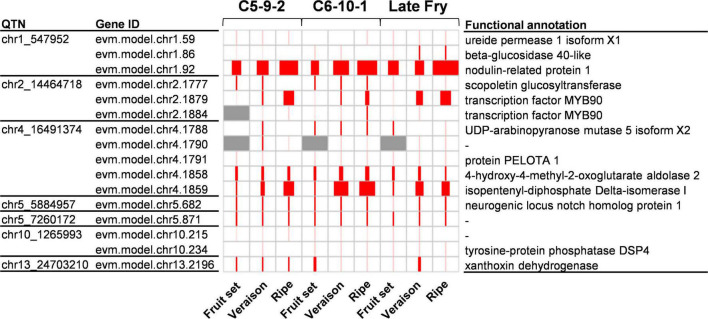
Expression of candidate genes identified by chr4_16491374. The QTN name and corresponding gene ID are listed on the left side of the panel. The functional annotation information is listed on the right side of the panel. In the panel, the size of red squares represents the TPM value of the corresponding gene. The max TPM was 1834.3.

To consider the case that the degradation pathway controls the quantitative property of antioxidants, we also investigated the candidate genes that are negatively correlated to the antioxidant activity. Among the 732 genes, a total of 16 genes showed significant negative correlations (*r* ≤ -0.7; *p* ≤ 0.05) by the Pearson correlation test (Figure 5). Among the 16 genes, 4-hydroxy-4-methyl-2-oxoglutarate aldolase identified by the chr4_16491374 QTN was the only gene related to the reduction of phenolic compound. The 4-hydroxy-4-methyl-2-oxoglutarate aldolase protein is involved in the degradation pathway of gallic acid in bacteria (Schomburg and Salzmann, 1990; Nogales et al., 2011). In a previous study using whole muscadine berries, gallic acid was highly correlated with DPPH and FRAP activities (Darwish et al., 2021). Therefore, the degradation of gallic acid by the 4-hydroxy-4-methyl-2-oxoglutarate aldolase protein is most likely involved in the quantitative properties of antioxidants, acting as a negative regulator.

### Quantitative trait nucleotides for antioxidants in muscadine berry skin

Although intensive genetic studies have been conducted with *Euvitis* species, no genetic studies related to antioxidant properties have been performed. In this study, we identified two highly significant QTNs, chr2_1446718, and chr4_16491374, associated with the TPC, and DPPH/FRAP capacities. The highest phenotypic variation of these QTNs was observed in DPPH activity at 8.25% (chr2_1446718) and 18.92% (chr4_16491374) (Table 2). These are the first QTNs for the quantitative properties of the antioxidant trait in muscadine grapes. Because we used the transferable markers in this study, the same markers would be applied to bunch grapes to study the QTNs for antioxidants.

With the two QTNs, the current study identified the positively correlated gene with the antioxidants, UDP-glycosyltransferase, and the negatively correlated gene, 4-hydroxy-4-methyl-2-oxoglutarate aldolase. The genetic studies of antioxidants have identified candidate genes in multiple crops by GWAS analysis. Interestingly, the GWAS results of antioxidants in barley, pearl millet, and common bean identified the UDP-glycosyltransferase gene as the candidate gene for the quantitative property of antioxidants (Han et al., 2018; Nadeem et al., 2020; Yadav et al., 2021). In apple, the GWAS result of a flavonoid, quercetin, identified the UDP-glycosyltransferase as the candidate gene (McClure et al., 2019). These facts support that the UDP-glycosyltransferase gene identified by the chr2_1446718 QTN in muscadine is also involved in the quantitative property of antioxidants.

In the case of the negatively correlated gene, 4-hydroxy-4-methyl-2-oxoglutarate aldolase, there was no similar finding in genetic studies for antioxidants in plants. The phenolic compounds are subject to rapid turnover and degradation (Barz and Hoesel, 1979). Our study also showed the rapid decline of the antioxidant capacities in muscadine berry skins between the Fruit-Set and Véraison stages (Figure 3). This profile may occur by natural degradation or innate degradation pathway. The degradation pathway of phenolic compounds in plants is not characterized yet. However, it is known that a soil bacteria, *Pseudomonas putida*, degrades gallic acid to 4-carboxy-4-hydroxy-2-oxoadipate, which is then cleaved by 4-hydroxy-4-methyl-2-oxoglutarate aldolase (Tack et al., 1972; Nogales et al., 2011). The identification of the 4-hydroxy-4-methyl-2-oxoglutarate aldolase homolog in the muscadine grape by a QTN associated with antioxidants may suggest the existence of the degradation pathway of phenolic compounds in plants. The negative correlation between the expression of the 4-hydroxy-4-methyl-2-oxoglutarate aldolase gene and the antioxidant properties during berry development also supports the contribution of 4-hydroxy-4-methyl-2-oxoglutarate aldolase in the negative regulation of antioxidant activity in muscadine. The identification of the negative regulator of antioxidants would be very useful in utilizing gene-editing technology for breeding. Therefore, further studies on this gene in muscadine grapes are needed.

## Conclusion

This study provides the first genetic evidence of the loci controlling the quantitative properties of antioxidant character in muscadine berry skin. Because the markers used in this study are transferable among *Vitis* species, the results would be applicable to various breeding populations in both *Euvitis* and *Muscadinia* grapes. Through the genetic study with transcriptome analysis, we identified two candidate genes, UDP-glycosyltransferase and 4-hydroxy-4-methyl-2-oxoglutarate aldolase, positively and negatively correlated to the quantitative property of antioxidant activity, respectively. The previous genetic studies of antioxidants from multiple crops also identified UDP-glycosyltransferase as a candidate gene. Therefore, the UDP-glycosyltransferase gene is a highly probable stimulator candidate for muscadine antioxidants. The negatively correlated gene was the homolog of the gallic acid degradation pathway gene of bacteria. However, further studies are needed because the degradation pathway of phenolic compounds in plants is unknown.

## Data availability statement

The datasets presented in this study can be found in online repositories. The names of the repository/repositories and accession number(s) can be found below: https://www.ncbi.nlm.nih.gov/, PRJNA775666 and PRJNA810835.

## Author contributions

MP and AD: conceptualization, methodology, investigation, validation, and formal analysis. MP: writing—original draft preparation, software, and visualization. RE, VT, and KS: writing—review and editing. IE-S: conceptualization, methodology, validation, formal analysis, resources, data curation, writing—review and editing, supervision, project administration, and funding acquisition. All authors approved the final manuscript.

## Footnotes

1
https://www.uniprot.org/

2
https://www.genome.jp/kegg-bin/show_organism?org=vvi
